# Concrete Mesostructure Modeling via Random Radius Field and Rigid Body Dynamics Packing

**DOI:** 10.3390/ma19061099

**Published:** 2026-03-12

**Authors:** Zhanbiao Zhang, Hui Wu, Mingzhuan Wei, Xiaogang Zhang, Yin Zhou, Xingyi Hu

**Affiliations:** 1 Technology Research & Development Center, The Seventh Engineering Co., Ltd. of China First Highway Engineering Co., Ltd. (CFHEC), Zhengzhou 451450, China; 2 School of Civil Engineering, Chongqing Jiaotong University, Chongqing 400074, China; 3 Department of Civil Engineering, KU Leuven, Campus Gent, 9000 Gent, Belgium

**Keywords:** mesostructure, concrete, random radius field, rigid body dynamics, recycled aggregate concrete (RAC)

## Abstract

This paper proposes a novel and efficient mesostructure generation framework integrating stochastic geometry with physically based packing. First, a random radius field (RRF) method is developed, utilizing multi-scale noise superposition and topology optimization to generate 3D aggregates with realistic and controllable morphologies. Second, a packing strategy based on Rigid Body Dynamics (RBD) is developed to simulate the physical casting process including gravity falling and vibration, achieving high-density aggregate skeletons. The framework is validated through the generation of a multi-phase mesostructure and the fracture simulation of recycled aggregate concrete (RAC). The simulation results successfully reproduced the crack propagation patterns and damage evolution paths associated with different aggregate shapes. These findings confirm the capacity and effectiveness of the proposed framework as a robust tool for the mesoscopic modeling of heterogeneous concrete materials.

## 1. Introduction

Concrete is a quite essential quasi-brittle composite material whose macroscopic mechanical behaviors—such as a non-linear stress–strain response, damage evolution, and fracture toughness—are intrinsically governed by its material heterogeneity. This heterogeneity is particularly pronounced at the mesoscale, where concrete is characterized as a multi-phase system comprising coarse aggregates, mortar matrix, and the Interfacial Transition Zone (ITZ). Given the complexity of these interactions, mesoscale numerical simulation has become an indispensable tool for bridging the gap between microscopic structure and macroscale performance [1,2,3], especially with the development of sustainable construction practice [4,5]. However, the predictive capability of these models is fundamentally dependent on the realistic representation of their mesostructures, specifically regarding aggregate geometry and particle packing models [6].

The fidelity of mesoscale mesostructures relies heavily on the accurate generation of particle geometry. In early numerical studies, aggregates were frequently simplified as idealized shapes, such as spheres, ellipsoids, or superballs, to ensure computational efficiency [7,8]. While widely used, these smooth shapes significantly simplified key morphological features, such as angularity and concavity, which underestimates the local stress concentration, fracture paths, and macroscopic strength [1,3]. To capture more realistic geometries, Voronoi-tessellation-based methods have been widely adopted to generate random polyhedral aggregates. When coupled with shrink–expand operations, these models can effectively improve realism and enable systematic variation of morphology indices (e.g., convexity) [9,10,11]. However, satisfying pre-described grading curves while achieving high packing densities with Voronoi polyhedra remains mathematically challenging [10]. Those methods mentioned above can be categorized as parametric method since all of them are based on the defined parameters to control the morphology of particles.

To represent the geometric features in a more realistic way, image-based approaches such as X-ray Computed Tomography (XCT) and 3D scanning with spherical harmonics were applied to reconstruct aggregate geometry and particle distribution from physical concrete samples [12,13]. It can directly reconstruct real aggregate shapes and builds the experimental–numerical one-to-one mappings [14], which are crucial for later mechanical and durability simulation. Despite their accuracy, these methods are equipment-intensive and high-computational resource demand, resulting in low efficiency compared to the parametric method [1,6,15]. Therefore, it is needed to propose a strategy to balance the generation efficiency and particle morphology representativeness in concrete mesostructure generation.

An aggregate packing strategy is another critical aspect to the mesostructure. The “Take-and-Place” method was the most widely used method during the early development of the field due to its simplicity. By “taking” the particles from the library and “placing” them in the defined domain one by one, this process utilizes geometric checks to ensure packing density and avoid overlap between adjacent particles. Wang et al. [7] extended these geometric schemes to 3D aggregates of various shapes, such as sphere, ellipsoid, and convex polyhedron. While it is effective for low-to-medium volume fractions (30–45%) and simple geometries, this method suffers from a “jamming limit,” where the successful rate of placement drops exponentially at high aggregate volume fractions (>60%) and complex particle geometries due to the much higher computational demand [1,6,16,17]. To overcome this, Random Walking Algorithms (RWAs) have been introduced in recent years. Unlike static placement, RWA dynamically translates and rotates particles to create space for subsequent particles, yielding significantly higher compactness [13,18]. Furthermore, physics-based strategies employing Discrete Element Methods (DEM) to pack the particles with realistic geometries by simulating physical interactions between particles such as gravity settlement and vibration during the packing process [19,20]. Traditional DEM-based simulations rely on a “soft-contact” approach, allowing slight particle overlaps to compute interaction forces and update motion states. Although it accurately models physical deformations and generates highly realistic packings, the computational cost becomes prohibitive for a large number of particles. Ultimately, performing such rigorous calculations is disproportionately expensive when the objective is to generate a geometric skeleton [15,21]. Table 1 gives a summarize of the recent mesostructure generation method.

To overcome the dichotomy between morphological fidelity and computational efficiency, this paper proposes a comprehensive framework that integrates a novel random radius field (RRF) generation method with a physically based packing strategy. On the geometric level, the proposed technique uses RRF to produce realistic particle geometry without the computational overhead of image-based reconstruction. On the packing level, we utilize an efficient Impulse-Based Rigid Body Dynamics (I-RBD) engine that achieves a high packing density while significantly mitigating the computational costs compared to traditional Discrete Element Methods (DEMs). This approach enables the rapid formation of a dense, interlocked aggregate skeleton that fully accounts for complex particle morphologies. The effectiveness of this framework is validated through the generation and fracture simulation of Recycled Aggregate Concrete (RAC), demonstrating its potential as a robust and efficient platform for virtual material testing.

## 2. Particle Generation

**Figure 1:**
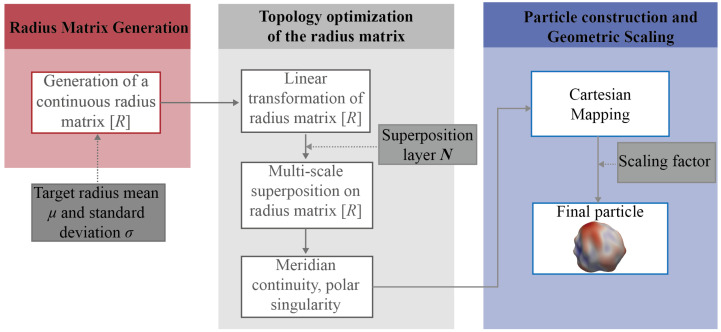
Diagram of the particle generation.

Within this section, a new particle geometry generation method was proposed using the RRF theory. As indicated in Figure 1, the whole generation procedure includes 3 main steps: Radius matrix generation, topology optimization of the radius matrix, and particle surface construction and geometry scaling.

### 2.1. Radius Matrix Generation

Following established methodologies, the closed surface of a concrete aggregate particle can be mathematically represented by a radius function $r_{\left(\theta ,\phi \right)}$ in a spherical coordinate system, where θ and ϕ denote the polar and azimuth angles, respectively. In the numerical simulation, this continuous function is discretized into a radius matrix, *R*, as shown in Equation (1).

(1) $$R=\left[\begin{array}{ccc} r_{\left(\theta_{1},\phi_{1}\right)} & \cdots & r_{\left(\theta_{1},\phi_{n}\right)}\\ \vdots & \ddots & \vdots \\ r_{\left(\theta_{n},\phi_{1}\right)} & \cdots & r_{\left(\theta_{n},\phi_{n}\right)} \end{array}\right]$$

**Figure 2:**
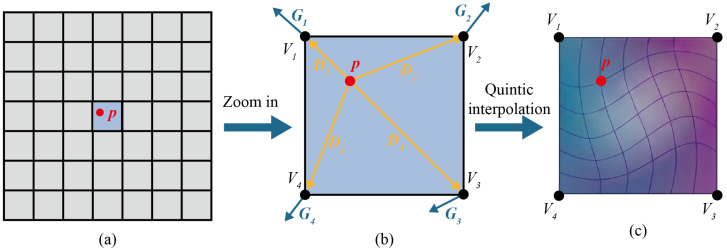
Diagram of the radius matrix generation: (**a**) 2D grid; (**b**) gradient vector and distance vector; (**c**) Interpolation.

Therefore, the primary challenge of particle surface generation is to construct a radius matrix, *R*. Specifically, the radius matrix has a certain degree of randomness to reflect the random morphology of the particle surface, but on the other hand, it is not completely random, as any two neighboring elements in different directions are correlated in order to form a continuous surface. Based on this feature, this paper proposes a novel interpolation method for generating a spatially correlated random radius matrix, *R*. The generation process involves a 2D interpolation method on a regular grid, which is subsequently mapped to the spherical domain. The details are explained below and indicated in Figure 2:

(1) Grid matrix creation: A 2D regular grid that covers the domain of the intended radius matrix, *R*, is created. For any point, *p*, in the target matrix, *R*, it can be located within a unit element of this grid, as shown in Figure 2a.

(2) Gradient vector ($\left\{G\right\}$) generation: a random unit gradient vector is assigned to each vertex of *R* to define the local slope of the matrix (indicated as the blue arrows in Figure 2b).

(3) Distance vector ($\left\{D\right\}$) computation: For any internal point, p(x,y), within a grid cell, find its 4 adjacent grid vertices, and compute the distance vectors from *p* to these four surrounding vertices, as indicated with the orange arrows in Figure 2b.

(4) Weight ($\left[W\right]$) calculation: The scalar influence of each vertex on point, *p*, is determined by the dot product of gradient and distance vectors: $\left[W\right]=\left\{G\right\}\cdot \left\{D\right\}$, which is used as the weight factor of each grid vertex to point *p*;

(5) Interpolation: The final value at point *p* is then obtained by interpolating the weights of the four vertices $\left[W\right]$. To obtain a naturally smooth transition between grid cells, a quintic interpolation function is utilized as shown in Figure 2c:

(2) $$f\left(t\right)=6t^{5}-15t^{4}+10t^{3}$$

**Figure 3:**
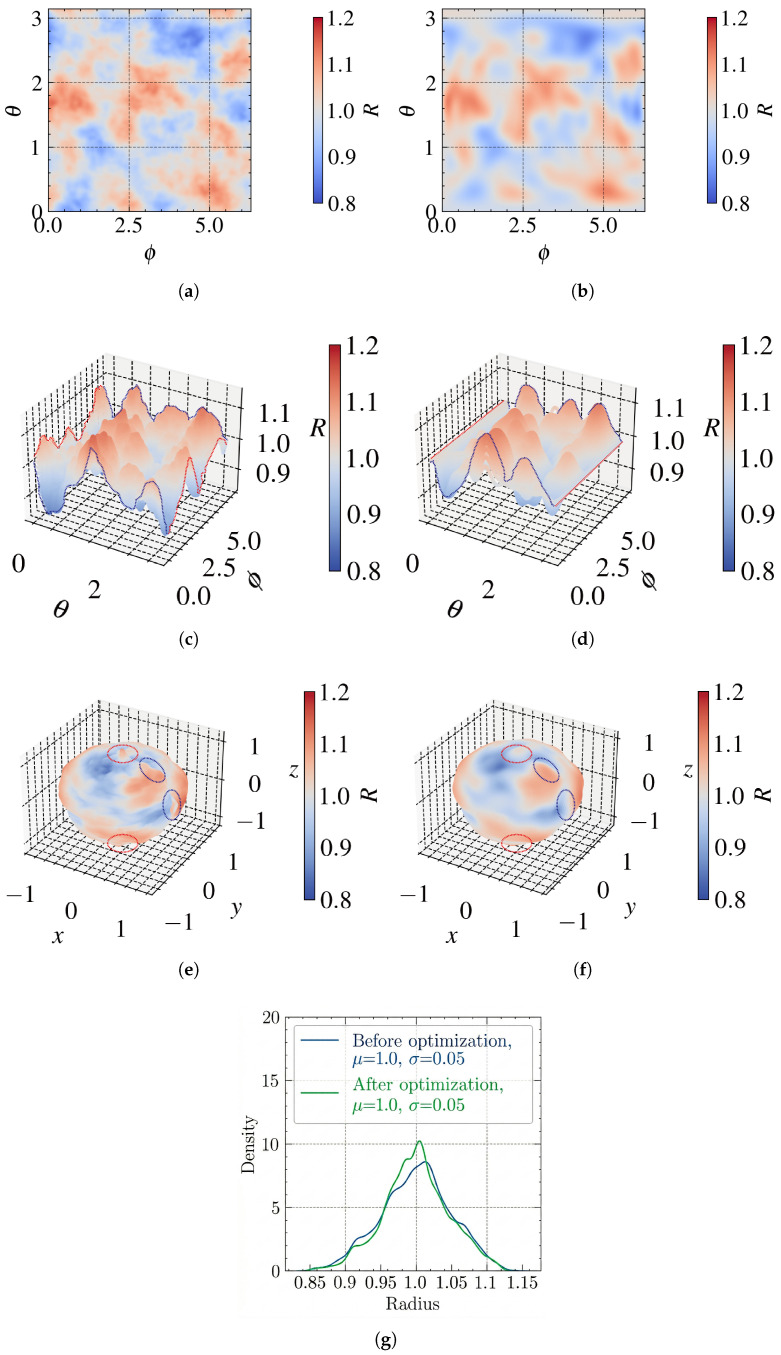
Radius matrix (μ=1.0, σ=0.05) and corresponding 3D surfaces before and after optimization: (**a**,**b**) matrix grids in 2D; (**c**,**d**) matrix in 3D; (**e**,**f**) 3D surfaces; (**g**) comparison of radius distributions.

By repeating steps (2)–(5) above for each internal point *p* within the matrix cells, a radius matrix, *R*, can be obtained. This matrix serves as the “morphological gene” of the particle, capturing the random yet smooth texture characteristic of natural aggregates (NAs). As indicated in Figure 3a–c, an example of the generated radius obtained based on the proposed method is visualized in 2D and 3D, respectively. It is obvious that the resolution of the grid matrix significantly influences the resulting particle morphology. A higher resolution enhances the representation of surface features, thereby improving morphological fidelity, but at the expense of an increased computational cost. Based on the sensitivity analyses reported in previous studies [1,24], a resolution of 80 × 80 is adopted in this work to achieve a balance between morphological accuracy and computational efficiency.

### 2.2. Statistical Transformation and Smooth of the Radius Matrix

#### 2.2.1. Statistical Transformation of the Radius Matrix

The raw radius matrix, *R*, generated by the above method consists of normalized noise values. To represent realistic aggregates, these values must be mapped to physical dimensions. Moreover, each element in *R* should be positive, since they represent the radius of each point on the surface. Most importantly, the statistical parameters of *R* are strongly correlated with the morphology of the resulting particle, as demonstrated in our previous study [17]. Therefore, a linear transformation is performed on *R* to adjust the statistical distribution of *R*, as in Equation (3):

(3) $$R_{tar}=\frac{\sigma }{\sigma_{R}}*\left(R-\mu_{R}\right)+\mu$$

where σ and μ are the target standard deviation and the mean value of all radii in target $R_{tar}$, respectively, while $\sigma_{R}$ and $\mu_{R}$ are the current standard deviation and the mean value of all radii in *R*, respectively. As discussed in ref. [17], a greater μ results in a larger particle, while σ indicates the amplitude of surface fluctuations, which is highly related to the angularity of the resultant particle. The parameters μ and σ can be either user-defined or derived from real particle data, allowing the generated particles to exhibit volumetric characteristics and angularity comparable to those of real particles.

While the linear adjustment of the statistics of *R* (μ,σ) effectively governs the overall morphology of the resultant particle, it is inherently limited in reproducing the complex surface texture since the single-frequency radius field tends to yield a simple and continuous surface. To enhance the surface texture (roughness), a multi-scale superposition strategy is proposed in this study: the final radius matrix $R_{final}$ is constructed by superimposing multiple independent radius matrix layers generated using the similar strategy described in Section 2.1, mathematically expressed as follows:

(4) $$R_{final}=\sum_{k=1}^{N}\frac{1}{2^{k-1}}P\left(R_{k}\right)$$

where *N* is the total number of superimposed layers, $R_{k}$ represents the $k^{th}$ radius matrix layer with its own statistical properties, and P represents the base Perlin noise generation function. Mathematically, this superposition introduces localized surface roughness by overlaying high-frequency disturbances onto the base geometry. Consequently, fine-scale surface irregularities are enhanced without compromising the overall volumetric stability governed by the primary low-frequency radius matrix layer. This approach effectively captures a wider range of surface textures, thereby enhancing the morphological realism of the generated particles. Based on repeated trials conducted by the authors, a value of N=5 is recommended, as it effectively reproduces the characteristic surface textures of the particles. The detailed analysis will be discussed in Section 2.4.

#### 2.2.2. Topology Optimization of the Radius Matrix

The final 2D radius matrix serves as a discretized unfolded map of the particle surface. To construct a watertight 3D geometry, a ”spherical mapping” has to be performed on this radius matrix. However, this process may introduce topological challenges at the boundaries (shown in Figure 3e), specifically the gaps along the meridian (where ϕ=0 and ϕ=2π) and the discontinuities on the poles (where θ=0 and θ=π). To eliminate topological challenges, the following optimizations are applied to the radius matrix $R_{final}$ before the final surface has been constructed:

(1) Meridian continuity. The radius values at the left and right boundaries of the grid must be identical to form a closed loop (as shown with the blue dashed line in Figure 3c,d).

(5) $$r_{\left(\theta_{i},0\right)}=r_{\left(\theta_{i},2\pi \right)}$$

(2) Polar singularity. All points along the top edge (θ=0) must converge to a single North Pole vertex, and similarly for the bottom edge (θ=π) to the South Pole. As shown with the red dashed line in Figure 3c,d, this is achieved by averaging the radius values along the respective rows:

(6) $$\begin{array}{cc} r_{\left(0,\phi_{j}\right)} & =\frac{1}{n}\sum_{j=1}^{n}r_{\left(\theta =0,\phi_{j}\right)}\\ r_{\left(\pi ,\phi_{j}\right)} & =\frac{1}{n}\sum_{j=1}^{n}r_{\left(\theta =\pi ,\phi_{j}\right)} \end{array}$$

By the above algorithm, the radius matrix is updated. However, such a surface only results in a closed surface, which still needs to be improved near the “zero-degree longitude” line since Equation (6) simply sets the corresponding values equal. A specialized interpolation algorithm [15] is applied to the pole regions to smooth out any derivative discontinuities, ensuring that the “seam” is visually and numerically smooth in the final 3D mesh.

A comparison between the example radius matrix (μ = 1.0, σ = 0.05) before and after the topology optimization is indicated in Figure 3. From Figure 3e,f, it can be observed that the north and south poles of the particle have been smoothed (indicated in the red circles), and the entire particle surface has been closed (indicated in the blue circles). Overall, the optimization has efficiently set the meridian and polar in the correct values to ensure a watertight and smooth particle surface. The radius distribution comparison shown in Figure 3g exhibits a similar distribution pattern with identical μ and σ values. This indicates that the optimization process preserves the statistical parameters of *R* and, therefore, does not alter the overall volume or angularity of the resulting particle.

### 2.3. Particle Surface Construction and Geometry Scaling

Once the finalized radius matrix, *R*, is obtained after all the transformation and optimization processes, the 3D surface mesh of the particle can be constructed via a Cartesian mapping process:

(7) $$\begin{array}{cc} x_{ij}= & r_{ij}\times \sin\left(\theta_{i}\right)\times \cos\left(\phi_{j}\right)\\ y_{ij}= & r_{ij}\times \sin\left(\theta_{i}\right)\times \sin\left(\phi_{j}\right)\\ z_{ij}= & r_{ij}\times \cos\left(\theta_{i}\right) \end{array}$$

This transformation generates a closed and “watertight” base particle with the desired size, angularity and surface texture (roughness) defined by the statistical properties radius matrix *R*. Because of the previous processes, this Cartesian mapping analytically maps the continuous 2D radius grid onto a closed spherical domain, it mathematically prevents the formation of open boundaries; therefore, the resultant particle surface should always be a closed one. Examples of the generated base particle using the proposed method are presented in Figure 3e,f. It should be noted that the base particle exhibits a quasi-spherical global aspect ratio, owing to the isotropic nature of the radius grid. To generate particles with a broader range of morphologies (e.g., flattened or elongated forms), a scaling transformation governed by the Elongation Index (EI) and Flatness Index (FI) can be applied to obtain particles with the desired aspect ratios.

### 2.4. Application Example

**Figure 4:**
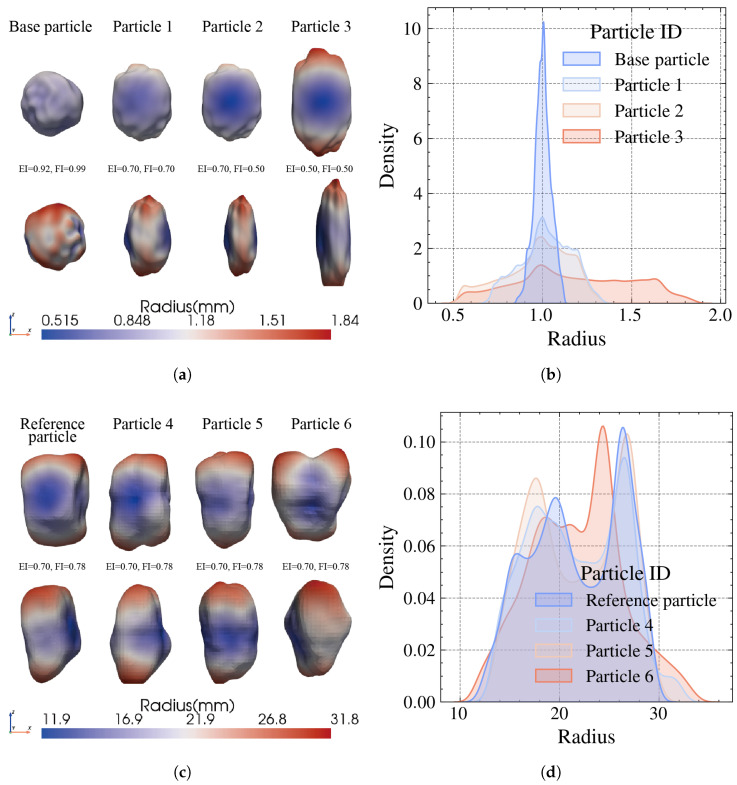
(**a**) Particles with user-defined parameters: μ = 1.0, σ = 0.05. (**b**) The corresponding radius distributions of the particles. (**c**) Particles with paramaters from real particle: μ = 21.8, σ = 4.2. (**d**) The corresponding radius distributions of the particles.

To demonstrate the capability of the proposed RRF method, a series of aggregates with distinct morphological characteristics were generated. In the first demonstration, the necessary parameter of the radius matrix were defined by the authors as follows to obtain the base particle mesh: μ = 1.0, σ = 0.05, and the number of superimposed layers *N* = 5. As shown in Figure 4a, the base particle exhibits a quasi-spherical geometry (EI=0.92, FI=0.99) with a concentrated radius distribution peaked around the mean value (blue histogram in Figure 4b).

By systematically reducing the aspect ratio indices (e.g., EI and FI) from 0. 9 to 0.5, the particle is gradually stretched and flattened to represent the diversity of particle shape form, as shown in Figure 4a. For instance, “Particle 3” (EI=0.50,FI=0.50) evolves into a highly elongated, flat aggregate. Figure 4b quantitatively captures the differences in radius distribution. While the base particle shows a narrow, sharp probability density function, the scaled particles exhibit progressively wider distributions.

To further validate the feasibility of the proposed method, a real particle obtained from 3D scanning was taken as a reference (shown in Figure 4c), and the statistical parameters of it are: μ = 21.8, σ = 4.2. And its aspect ratios are EI = 0.70 and FI = 0.78. Applying the proposed method, three particles are generated as shown in Figure 4c, while the radius distributions of them are compared in Figure 4d. The morphological characteristics of those particles are compared in Table 2. Overall, the proposed method provides an efficient framework for generating aggregates with arbitrary dimensions and morphological characteristics governed by predefined statistical parameters. The input parameters can be derived either from measurements of real particles or specified directly by the user. This approach enables the construction of a versatile and realistic particle library for subsequent packing simulations.

## 3. Particle Packing Strategy

In this section, a novel particle packing strategy is proposed based on an Impulse-based Rigid Body Dynamics (I-RBD). Unlike the traditional geometric packing strategies, the proposed method explicitly accounts for the physical laws governing gravity-driven free falling, collision, and bouncing to simulate the actual compaction and vibration processes involved in concrete casting. Additionally, this approach is able to achieve a high aggregate volume fraction, even for particles with highly irregular morphologies.

### 3.1. Physical-Based Theory

Unlike geometric packing algorithms (e.g., RWA) that rely on heuristic placement, the proposed method treats aggregates as discrete rigid bodies. The packing process utilizes the physics engine employing the I-RBD-based deterministic solver, which resolves the motion of each particle via sequential time-stepping. At each discrete time step, Δt, the solver executes collision detection, constraint solving, and state integration. Consequently, the kinematic state (position and orientation) of each particle is iteratively updated until the assembly converges to a static equilibrium.

In particular, each individual aggregate is idealized as non-deformable rigid bodies. The motion of a particle *i* with mass $m_{i}$ and inertia tensor $I_{i}$ is governed by the Newton–Euler equations of motion. For the translational and rotational components, the dynamic equilibrium is defined as:

(8) $$\begin{array}{cc} F_{net} & =m_{i}\frac{dv_{i}}{dt}\\ \tau_{net} & =I_{i}\frac{d\omega_{i}}{dt}+\omega_{i}\times \left(I_{i}\omega_{i}\right) \end{array}$$

where $v_{i}$ and $\omega_{i}$ denote the linear and angular velocities, respectively. $F_{net}$ represents external forces (gravity) and constraint forces (collisions), while $\tau_{net}$ represents the resulting moment.

For each irregular particle, collisions are detected based on its exact geometry. A hierarchical collision detection framework is employed to ensure both efficiency and accuracy. First, an Axis-Aligned Bounding Box (AABB) is used to rapidly identify potential collision pairs of particles, thereby excluding non-interacting particles. Secondly, for the potentially colliding pairs, the exact contact points and penetration depths are computed using the Gilbert–Johnson–Keerthi (GJK) distance algorithm and the Expanding Polytope Algorithm (EPA). This two-stage strategy enables efficient and robust handling of complex geometries. Upon detecting a collision, the non-penetration condition is enforced as a velocity-level constraint within the solver.

Instead of calculating continuous contact forces as used in many FEM simulations, the physics solver employs a sequential impulse solver, which enables it to iteratively apply instantaneous constraint impulses, J, to the contacting bodies to instantly correct their velocities and prevent penetration:

(9) $$\Delta v=M^{-1}J$$

where $M^{-1}$ is the effective inverse mass matrix of the contact pair. The physical behavior of the contact interface is parameterized by the Coefficient of Restitution (*e*), which controls energy dissipation (bounciness), and the Coulomb Friction Coefficient (μ), which limits the tangential impulse to simulate surface roughness.

To update the kinematic state of the particles, the differential equations are discretized utilizing a Semi-implicit Euler integration scheme, favored for its energy conservation properties and stability compared to explicit methods. The state update at time t+Δt proceeds as follows:

(1) Velocity update. The solver first updates velocities by integrating external forces and applying the resolved constraint impulses:

(10) $$v\left(t+\Delta t\right)=v\left(t\right)+M^{-1}\left(F_{ext}\Delta t+J_{constraint}\right)$$

(2) Position update. The new positions and orientations are then derived using the updated velocities:

(11) x(t+Δt)=x(t)+v(t+Δt)Δt

By iterating this physics pipeline, the trajectory of every aggregate is deterministically calculated, allowing the system to naturally evolve from a loose skeleton to a dense, interlocked packing state under gravity and vibration. It should be noticed that the proposed I-RBD strategy-based packing method is superior to traditional “soft-contact” DEM simulation in efficiency. Traditional DEM formulations employ “soft-contact” models between two particles, in which interaction forces are computed from microscopic overlaps between particles. This approach requires small time steps to resolve high-frequency elastic oscillations and ensure numerical stability. In contrast, the I-RBD strategy adopts a “hard-contact” formulation, in which contact constraints are enforced through instantaneous velocity impulses. By bypassing the explicit computation of transient elastic deformations, this framework permits substantially larger time steps and enables rapid convergence toward static equilibrium. Moreover, whereas conventional DEM frequently approximates particle geometry using sphere clumps, the proposed method operates directly on high-fidelity triangular meshes, accurately capturing the mechanical interlocking induced by realistic geometry of particles. Certainly, this method does not have the same level of computational precision as traditional DEM. However, considering the main objective is to obtain a compact and interlocked aggregate skeleton, this approach provides an effective balance between computational efficiency and the accuracy required to represent the governing physical interactions.

### 3.2. Particle Packing Process

**Figure 5:**
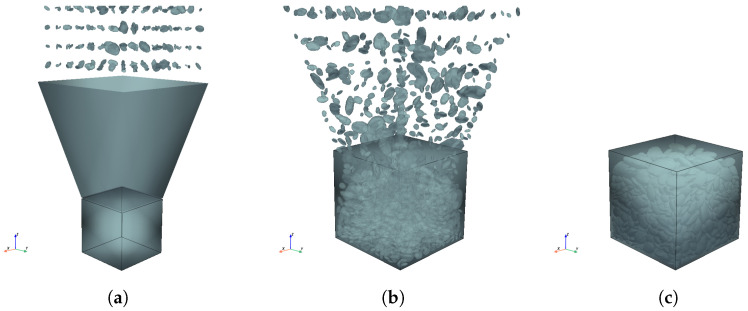
Physical packing procedure: (**a**) system setup and particle preparation; (**b**) gravity settlement; (**c**) vibration compaction.

Based on the theory outlined above, the packing process is performed as follows. The workflow simulates the actual casting procedure of concrete in a laboratory, including three main phases: particle preparation, gravity settlement, and vibration compaction. The detailed steps are illustrated in Figure 5.

(1) System setup and particle preparation. Before the simulation starts, the boundary conditions and material parameters are defined as follows. A rigid container (eg, a cube) is created to represent the concrete mold used in experiments. It is set as a “Passive” rigid body, meaning it remains stationary and acts as a fixed boundary for the aggregate packing. The aggregate particles generated in Section 2 are imported into the system. They are set as “Active” rigid bodies with calculated mass, allowing them to move, rotate, and collide under physical laws defined in Section 3.1. Two key parameters are assigned to simulate the mechanical behavior of particles: (1) friction (μ)—a friction coefficient is applied to simulate the rough surface of aggregates; (2) bounciness (*e*)—a low value is adopted to ensure the particles dissipate energy rapidly and do not bounce excessively upon collision, which might cause contact instabilities. The recommended values of these two parameters are summarized below based on the numerical trial-and-error experiments from the authors: μ∈[0.4,0.6] and e∈[0.05,0.2]. As shown in Figure 5a, particles are arranged in a loose grid suspended above the mold. Their orientations are randomized to ensure random distribution. To guarantee that every particle falls into the container correctly, a slideway is also created on top of the container, as can be seen in Figure 5a.

(2) Gravity settlement. Then, gravity ($g=9.81\;m/s^{2}$) is enabled on all the particles, resulting in free falling into the mold, colliding with the bottom and each other. After the falling stops, the particles form a loosely packed skeleton (Figure 5b). Due to the inter-particle friction and irregular shapes, large voids are formed in the skeleton, similar to the loose bulk state of aggregates.

(3) Vibration compaction. To eliminate the large voids and consolidate the aggregates, a vibration process is introduced by applying a continuous harmonic sinusoidal displacement to the mold. For any axis in i=X,Y,Z, the positional disturbance ΔP(t) at time *t* is defined as:

(12) $$\Delta P_{i}\left(t\right)=A_{v}\cdot \sin\left(2\pi f_{v}\cdot t+\psi_{i}\right)$$

where $A_{v}$ is the amplitude, $f_{v}$ is the vibration frequency, and $\psi_{i}$ is the phase offset on axis *i*.

This vibration reduces inter-particle friction, effectively breaking the arching structures. Consequently, the aggregates are allowed to rapidly rearrange and settle into a denser, physically realistic configuration (Figure 5c). The procedure runs until the kinetic energy of the system drops to nearly zero, indicating a stable equilibrium. Finally, the coordinates and rotation of all particles are exported to reconstruct the final mesoscopic model.

It is important to clarify the inherent idealizations of the proposed I-RBD packing strategy. While actual concrete casting is strongly governed by fresh-state rheology—such as paste viscosity, batching procedures, and specific compaction methods—simulating the complex fluid dynamics of the cement slurry is beyond the scope of this study. Instead, the primary objective is to overcome the computational bottlenecks of traditional particle-packing algorithms. By idealizing the viscous dampening of the mortar matrix and relying on hard-contact rigid-body dynamics, the framework deliberately trades rheological fidelity for computational efficiency. Ultimately, this approach successfully yields the dense, mechanically interlocked, and high-volume-fraction geometric skeletons required for subsequent mesoscale fracture simulations.

### 3.3. Application of the Packing Strategy

**Figure 6:**
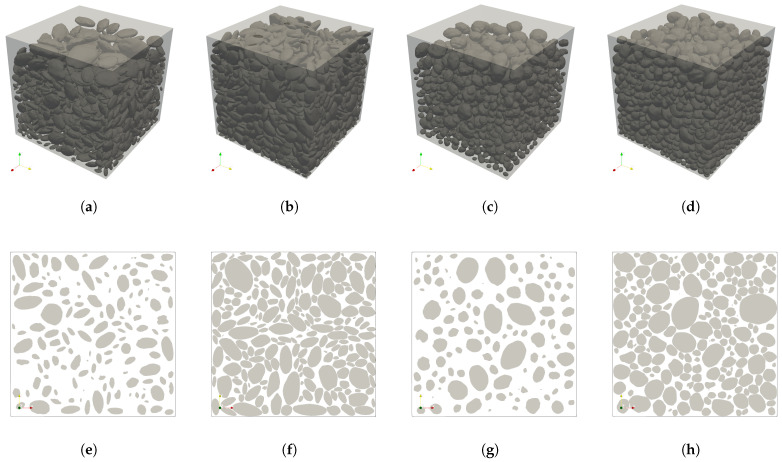
Visualization of packing models. 3D models: (**a**) flat, $f_{agg}$ = 40%; (**b**) flat, $f_{agg}$ = 70%; (**c**) round, $f_{agg}$ = 40%; (**d**) round, $f_{agg}$ = 70%. 2D models: (**e**) flat, $f_{agg}$ = 40%; (**f**) flat, $f_{agg}$ = 70%; (**g**) round, $f_{agg}$ = 40%; (**h**) round, $f_{agg}$ = 70%.

To demonstrate the robustness and versatility of the proposed I-RBD packing strategy, a systematic series of numerical experiments was conducted. Specifically, two groups of aggregates with distinct morphological characteristics were generated based on the target particle size distributions: “rounded” particles (EI=0.85,FI=0.75) and “flat” particles (EI=0.70,FI=0.50), with the required parameters of μ = 1.0, σ = 0.05, and *N* = 5. After the base particles were obtained, they were scaled into the target aspect ratio (EI and FI) and size fraction according to the Particle Size Distribution (PSD) data reported in ref. [25]. These particle sets were then subjected to the physical packing workflow. To evaluate the packing performance, the physical workflow was executed to achieve two distinct target coarse aggregate volume fractions ($f_{agg}$) for each morphological group (e.g., a moderate $f_{agg}$ = 40% and a high $f_{agg}$ = 70%). Consequently, four mesostructural models were successfully generated, as is visualized in Figure 6.

The computational efficiency of the entire framework was evaluated on a standard desktop workstation (Intel Core i9 CPU, 32 GB RAM, NVIDIA GeForce RTX 3060Ti, 8GB VRAM). The quantitative runtime for the full pipeline—including geometry generation, physical packing, and volumetric meshing via TetGen [26]—is summarized in Table 3. The most computationally demanding scenario—the high-density flat particle model (model b: 3322 particles, 69.7% volume fraction)—required 120 s for generation, 710 s for packing, and 492 s for meshing (yielding 301,097 elements). Notably, the physical packing stage remains the primary computational bottleneck due to the intense $O(n^{2})$ broad-phase collision detection required at elevated densities. Nevertheless, the ability to generate, pack, and mesh thousands of high-fidelity irregular particles in under 25 min signifies a substantial acceleration compared to traditional force-resolved DEM approaches.

These results highlight the superior performance of the proposed method above, demonstrating its ability to rapidly generate mesostructures with high aggregate volume fractions containing thousands of particles in minutes. Crucially, the resulting aggregate skeletons ensure physical realism with zero overlaps and distinct mechanical interlocking, thereby providing a robust geometric basis for subsequent mechanical simulations.

## 4. Framework Application and Mechanical Simulation of Recycled Aggregate Concrete

To demonstrate the capacity of the whole framework proposed in Section 2 and Section 3, this section applies it to the mesostructure generation of a multi-phase composite—recycled aggregate concrete (RAC)—followed by mechanical fracture simulations.

### 4.1. Mesostructure Generation and Discretization

The workflow of mesostructure generation follows a “bottom-up” generation sequence. First, the natural aggregates (NAs) are generated using the proposed stochastic radius field strategy; subsequently, these NAs are transformed into recycled aggregates (RAs) by appending a layer of AM, and finally, the dense packing algorithm discussed in Section 3 is employed to construct the full concrete mesostructure.

In the context of the geometric framework, the mesostructure of RAC is idealized as a five-phase composite system. The phases are defined based on their generation sequence. (1) Natural aggregate (NA): the core inclusion, generated directly by the algorithms in Section 2, representing the irregular aggregates. (2) Adhered mortar (AM): A porous coating layer attached to the NAs. The combination of NA and AM constitutes the recycled aggregate (RA). (3) New mortar (NM): the continuous phase filling the remaining volume. (4) The Old Interfacial Transition Zone (ITZ): the weak interface explicitly modeled between the AM and NA. Finally, (5) the New ITZ: the weak interface between NM and AM. This explicit representation of five phases aligns with state-of-the-art RAC mesoscale frameworks [1,6], enabling the failure mechanisms of the old and new interfacial zones to be captured.

A standard cubic RAC specimen with a side dimension of 100 mm is defined as a representative volume element (RVE), consistent with the common dimensions used in many RAC experimental studies and the Chinese natural standard [27] for a mechanical property test. Furthermore, because the chosen RVE dimension is four times the maximum particle size (25 mm), it satisfies established mesoscale criteria for ensuring statistically representative macroscopic behavior, thereby minimizing size-effect scatter across different random spatial realizations. To validate the mechanical response, the RAC model in this study is fully recycled aggregate concrete; e.g., the replacement ratio of RA is 100%, while the adhered mortar content ($R_{AM}$, defined as $R_{AM}=V_{AM}/V_{RA}$) is 30%, which represents a typical value reported in extensive experimental characterizations of RAC [6,28].

**Figure 7:**
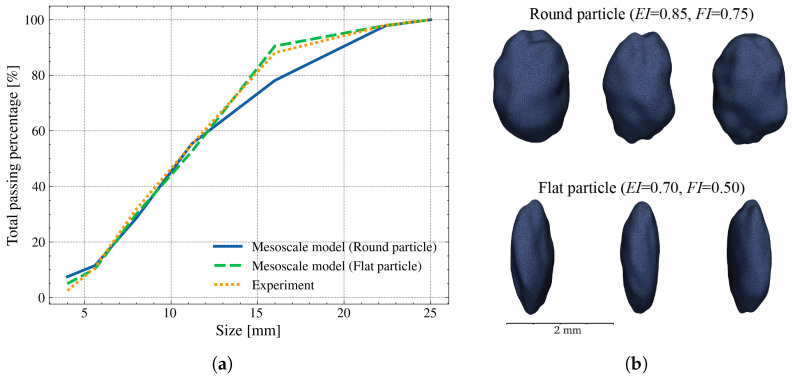
(**a**) Particle size distribution of RA in modeling and experiment; (**b**) particle examples in mesostructures.

The particle size distribution of RA is designed according to the experimental data [25], with a maximum size of 25 mm and the total volume fraction of 41.2%. Regarding particle morphology, this simulation directly adopts the same aggregate groups whose geometric indices were detailed in Section 3.3—flat (EI = 0.70, FI = 0.50) and round (EI = 0.85, FI = 0.75) particles. Figure 7a,b indicates the comparison of the PSD and the corresponding particle morphology. A total of 1631 and 1983 RA particles are generated for flat and round particles, respectively.

**Figure 8:**
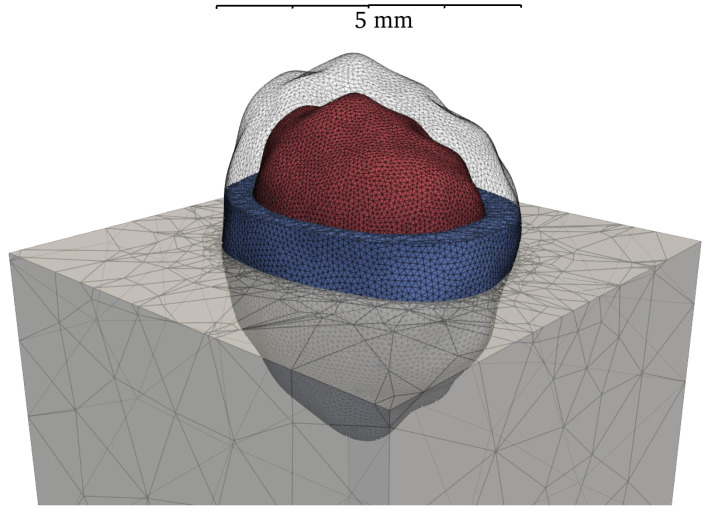
Diagram of adhered mortar generation (Red—NA; Blue—AM; Grey—NM).

In this study, the AM phase is produced by scaling up the NA and defining the evenly wrapped layer between this expanded geometry and the inner NA core as the AM on each particle, as indicated in Figure 8. The thickness of this layer is governed by the adhered mortar content ($R_{AM}$), which is determined by the linear scaling factor, *S*:

(13) $$S=\sqrt[{3}]{\frac{V_{RA}}{V_{NA}}}=\sqrt[{3}]{\frac{V_{RA}}{V_{RA}-V_{AM}}}=\sqrt[{3}]{\frac{1}{1-R_{AM}}}$$

With the substitution of $R_{AM}=0.3$, the required scaling factor *S* is 1.126 in this study.

To construct a dense and realistic granular skeleton, the generated RA followed a packing process based on the strategy discussed in Section 3. During the 20–80% packing time, the mold is subjected to a vibration function ($\Delta P\left(t\right)=0.6\times \left(4\pi \dot{t}+\frac{\pi }{12}\right)$) on *X* and *Y* directions.

**Figure 9:**
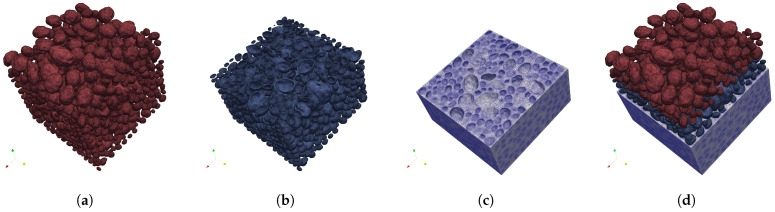
Tetrahedron meshes of the mesoscale model with round RA: (**a**) NA; (**b**) AM; (**c**) NM; and (**d**) RVE.

**Figure 10:**
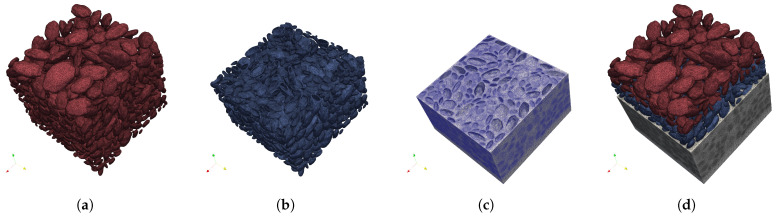
Tetrahedron meshes of the mesoscale model with flat RA: (**a**) NA; (**b**) AM; (**c**) NM; and (**d**) RVE.

The final step translates the triangulated mesh surfaces in STL format into a volumetric finite element mesh using TetGen 0.8.2 [26]. The material phases were meshed using 4-node linear tetrahedral elements, and the ITZs were modeled using zero-thickness cohesive elements. An adaptive meshing approach was applied to balance computational efficiency with numerical accuracy. Specifically, a larger maximum element size of 5.0 mm was applied within each material phase (i.e., the NA and NM phases). Conversely, a refined local mesh size of 1.0 mm was enforced at the phase boundaries and complex interfacial regions, encompassing the AM and the ITZs. This adaptive strategy ensures that at least one layer of elements explicitly represents the AM thickness while restricting the cohesive element size to remain below the characteristic fracture length of the interfaces. Figure 9 and Figure 10 illustrates the finite element model with round and flat RA.

### 4.2. Mechanical Simulation of RAC

Based on the reconstructed mesoscopic finite element model, a uniaxial compression simulation is performed to investigate the failure mechanism of RAC. To accurately capture the non-linear damage evolution of RAC, distinct constitutive models and the corresponding parameters are assigned to the different material phases. The selection of them is grounded in the previous mesoscale modeling studies of RAC [1,6] and the calibration protocols established from the existing experimental data [20,28]. The specific mechanical parameters adopted for all phases are summarized in Table 4.

In accordance with widely accepted mesoscale modeling practices, the NA phase is typically idealized as an elastic since its significantly higher strength relative to the surrounding matrix. In contrast, the cementitious phases, including the NM and AM, exhibit quasi-brittle behavior characterized by strain softening, which has been assigned with Concrete Damage Plasticity (CDP) model in ABAQUS. However, the mechanical properties of AM is weaker than NM reported in many statistical data from literature. Therefore, it is assigned 70% mechanical properties relative to the NM, which is a calibrated reduction factor supported by nano-indentation and micro-mechanical experimental literature [1,28]. The final values of them are calibrated by matching the experimental compression ϵ−σ curve to the simulated curve. To explicitly capture interfacial damage and crack initiation, this region is modeled using Zero-thickness Cohesive Elements governed by a traction-separation law, while the material parameters of old ITZ and new ITZ were set to approximately 70% of the properties of AM and NM, respectively, according to the intensive literature review of the previous mesoscale modeling studies [1].

The simulation setup mimics a standard displacement-controlled uniaxial compression test on a cube. The bottom surface of the specimen is fully constrained ($U_{x}=U_{y}=U_{z}=0$) to represent a rigid platen. A uniform vertical displacement corresponding to a constant strain rate is applied to the top surface. To ensure a quasi-static response and minimize inertia effects within the explicit solver, the loading is applied using a smooth step amplitude curve over a duration of 0.1 s. The meshing strategy described in Section 4.1 results in a refined multi-scale mesh. The final model consists of approximately 2.8 million tetrahedral elements and 0.4 million cohesive elements.

**Figure 11:**
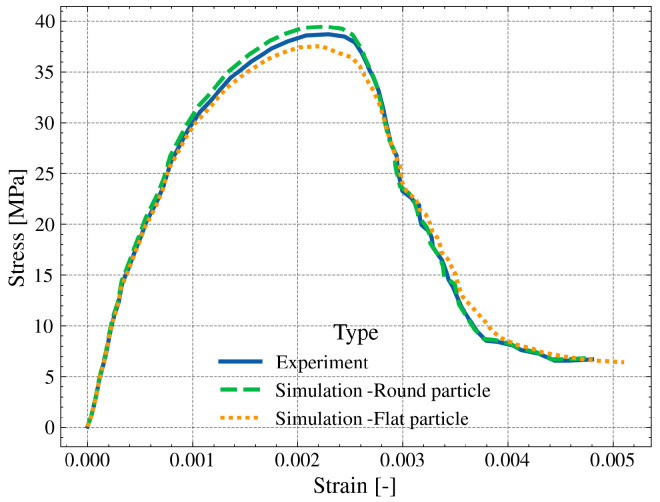
Simulation results of compressive strain–stress curves of RAC.

Figure 11 indicates the strain-stress curve of the corresponding simulations. Generally, the simulated stress-strain responses of the recycled aggregate concrete (RAC) models exhibit typical quasi-brittle behavior characteristic of cementitious composites, encompassing a linear elastic stage, a stable crack propagation stage (hardening), and a post-peak softening stage. However, the geometric morphology of the RA significantly governs the mechanical indices. The model comprising round particles exhibits relatively higher peak stress and elastic stiffness. This is attributed to the smooth surface curvature, which facilitates uniform stress distribution and mitigates local stress concentrations. In contrast, the model incorporating flat particles demonstrates a slight reduction in peak strength but an enhanced residual bearing capacity in the post-peak phase. This phenomenon is grounded in the “dual-effect” of the aggregate shape: while the sharp corners of flat aggregates induce severe stress concentrations that trigger earlier damage initiation (reducing peak load), their anisotropic geometry enhances the macro-geometric interlocking between the aggregates and the mortar matrix. This interlocking mechanism effectively provides an additional rotational constraint and frictional resistance during the softening stage, preventing the precipitous drop in stress observed in the simplified round particle model.

## 5. Conclusions and Discussion

### 5.1. Conclusions

This paper has proposed a novel framework for the generation of a concrete mesoscopic model, integrating an RRF particle generation method with a physically based particle packing strategy. The validity of the proposed framework has been demonstrated through the modeling and uniaxial compression simulation of RAC. The main conclusions are summarized as follows.

(1) Efficient generation of realistic geometries. The proposed RRF method, enhanced by topological optimization and multi-scale superposition, successfully reproduces the complex morphology of concrete aggregates. The framework enables the parametric generation of an aggregate with controllable shape indices (EI and FI), overcoming the limitations of simplified geometric primitives.

(2) High-density physically based packing. By applying a rigid body dynamic, the proposed packing strategy inherently accounts for gravity, collision, and mechanical vibration effects. The hard-contact formulation circumvents the substantial computational expense associated with traditional soft-contact DEM approaches and overcomes the “jamming limit” commonly encountered in geometric placement algorithms. As a result, it efficiently generates high-volume-fraction aggregate skeletons (≥70%) composed of thousands of highly irregular particles within minutes, while preserving realistic inter-particle interlocking.

(3) Framework validation through RAC Simulation. The application of the proposed framework to RAC confirms the capability to handle complex multi-phase systems (including NA, AM, NM, and ITZs). The simulation results successfully captured the influence of aggregate morphology on the macroscopic mechanical response.

### 5.2. Limitations and Future Perspectives

The primary contribution of the proposed framework lies in the capacity to efficiently generate realistic concrete mesostructures, with a particular emphasis on reproducing irregular particle morphologies and physically representative aggregate skeletons. To maintain computational efficiency at this scale, the framework incorporates necessary idealizations. Accordingly, the explicit modeling of microcracks, porosity, and fresh-state rheological behavior is beyond the present scope. However, future research will focus on incorporating these microstructural defects, thereby enhancing the physical fidelity of the mesostructures and enabling a more accurate representation of the concrete heterogeneity in mesoscopic simulations.

Furthermore, this paper has applied the proposed framework to the mechanical simulation of RAC at the material level under ambient, quasi-static loading conditions. The time-dependent phenomena—such as creep-induced restrained stresses [29]—as well as dynamic multi-physics interactions [30], are not considered in the present work. These behaviors will be important directions for future research.

Ultimately, the fundamental objective of mesoscale modeling of concrete material is to establish a mechanistic link between localized material degradation and macroscopic structural and durability behavior [31]. With ongoing advancements in computational efficiency and numerical implementation, the next stage of this research will focus on upscaling the framework to larger structural components (e.g., reinforced concrete members), thereby providing a platform for advanced structural health monitoring and macroscale inverse analysis [32].

## Figures and Tables

**Table 1:** Overview of recent methods in mesostructure generation: advances and limitations.

Aspect	Main Recent Advances	Key Limitations	Citations
Particle geometry generation	Parametric method: Circle, Ellipse, and Convex polygon; Voronoi-tessellation-based method. Image-based method: X-CT; 3D scanning and spherical harmonic reconstruction	Simplification of the morphology of particles; Balance between morphological realism and generation efficiency	[3,9,10,12,16,20]
Particle packing	“Take-and-Place”, Random Walking Algorithm, DEM, and hybrid algorithms.	Trade-offs between packing density, physical realism, and computational cost	[7,13,14,16,18,19,20,22,23]

**Table 2:** Parameters used in particle generation and corresponding morphological properties.

Particle ID	N	a	b	c	V	S	EI	FI
Base	5	2.183	2.014	1.994	4.294	13.262	0.922	0.990
1	5	2.610	2.000	1.533	3.918	12.989	0.766	0.766
2	5	2.610	2.000	1.095	2.799	11.201	0.766	0.547
3	5	3.654	2.000	1.095	3.918	15.055	0.547	0.547
Reference	5	55.58	39.15	30.36	37566.29	6164.17	0.704	0.775
4	5	57.65	40.53	31.17	37072.77	6019.60	0.703	0.769
5	5	58.09	40.66	31.65	35833.07	5801.33	0.700	0.778
6	5	55.08	40.73	31.94	42384.06	6639.05	0.739	0.784

**Table 3:** Performance metrics for different mesostructure packing models.

Mesostructure No.	$f_{\mathrm{agg}}$ [%]	Particle Number [-]	Generation Time [s]	Packing Time [s]	Meshing Time [s]	Mesh Number [-]
a	41.4	1942	72	544	386	209,426
b	69.7	3322	120	710	492	301,097
c	41.7	1631	63	410	376	187,423
d	70.3	2765	108	482	406	135,754

**Table 4:** Mechanical parameters in the mesoscale simulation.

Property Category	Natural Aggregate	New Mortar	Adhered Mortar	New ITZ	Old ITZ
Density, ρ (kg/$m^{3}$)	2650	2400	2200	-	-
Elastic Modulus, *E* (GPa)	70	25	18	-	-
Compression Strength, $f_{c}$ (MPa)	-	40	28	-	-
Tensile Strength, $f_{t}$ (MPa)	-	3.5	2.4	-	-
Elastic Stiffness, $K_{nn}$ (MPa/mm)	-	-	-	$10^{6}$	$10^{6}$
Cohesive Strength, $t_{n}$ (MPa)	-	-	-	2.45	2.00
Fracture Energy, $G_{f}$ (N/mm)	-	-	-	0.04	0.03

## Data Availability

The original contributions presented in this study are included in the article. Further inquiries can be directed to the corresponding author.

## Abbreviations

The following abbreviations are used in this manuscript:

AABB	Axis-Aligned Bounding Box
AM	Adhered Mortar
CDP	Concrete Damage Plasticity
DEMs	Discrete Element Methods
EI	Elongation Index
FI	Flatness Index
GJK	Gilbert–Johnson–Keerthi
I-RBD	Impulse-Based Rigid Body Dynamics
ITZ	Interfacial Transition Zone
NA	Natural Aggregate
NM	New Mortar
RA	Recycled Aggregate
RAC	Recycled Aggregate Concrete
RRF	Random Radius Field
RVE	Representative Volume Element
RWAs	Random Walking Algorithms
X-CT	X-Ray Computed Tomography
